# Adjunct cell therapy for hypoplastic left heart syndrome: therapeutic promise requires targeted design strategy

**DOI:** 10.1186/s13287-025-04717-4

**Published:** 2025-11-06

**Authors:** Christian P. Brizard, Salvatore Pepe

**Affiliations:** 1 Department of Paediatrics, University of Melbourne, Royal Children’s Hospital Melbourne, Melbourne, Australia; 2 Heart Research Group, Murdoch Children’s Research Institute, Royal Children’s Hospital Melbourne, Melbourne, Australia; 3 Department of Cardiac Surgery, Royal Children’s Hospital Melbourne, Melbourne, Australia

**Keywords:** Congenital heart disease, Hypoplastic left heart syndrome, Single ventricle, Umbilical cord blood, Mononuclear cells, Stem cell transplantation, Fontan palliation

## Abstract

Letter to the editor regarding: Gallego-Navarro C, Jaggers J, Burkhart HM, Carlo WF, Morales DL, Qureshi MY, Rossano JW, Hagen CE, Seisler DK, Peral SC, Nelson TJ. Autologous umbilical cord blood mononuclear cell therapy for hypoplastic left heart syndrome: a nonrandomized control trial of the efficacy and safety of intramyocardial injections. Stem Cell Res Ther. 2025 May 1;16(1):215. doi: 10.1186/s13287-025-04316-3. This phase IIb clinical cell therapy trial with autologous cord blood mononuclear cells is notable and highly commended as an important effort in very challenging clinical conditions in which new cell treatment advancements have been limited. In light of their findings of unfavourable clinical findings, our letter highlights that their study is intertwined with the complexity of the clinical pathology within immature pediatric hearts, cardiac surgical intervention strategy and other related study design hurdles. These difficulties contribute to confound the interpretation of cell treatment efficacy and clinical safety. Based on this study and our own recent clinical study, we make a call for future studies testing cell therapy in the congenital heart disease setting, to implement far greater consideration of the mode and timing of cell delivery and congruency with myocardial tissue remodelling and developmental growth processes in small hearts.

Recently published in *Stem Cell Research & Therapy* [1], the phase IIb clinical trial by Gallego-Navarro et al. heralds an important milestone in the pursuit of stem cell therapies for congenital heart disease. As the largest stem cell trial to date in hypoplastic left heart syndrome (HLHS), it provides critical data on the efficacy and safety of autologous umbilical cord blood mononuclear cells (CB-MNC) during stage II surgical palliation. The investigators should be commended for successfully coordinating this complex multicentre trial. With 95 children enrolled across 9 institutions, it provides robust data on the safety profile of CB-MNC therapy. The use of a control group, though non-placebo and non-randomized, facilitates more meaningful comparisons than previous uncontrolled studies.

Despite considerable work, the results fail to demonstrate a benefit in cardiac function, leaving open questions regarding safety and efficacy of cell therapy implemented by intramyocardial injection into the univentricular myocardium. Significant limitations stand out. Firstly, the non-randomized design introduces potential selection bias imbalance across groups due to variation in anatomical anomalies and intrinsic severity. Secondly, assessment of right ventricular (RV) function by echocardiography rather than cardiac MRI for the primary endpoints is subject to operator-related variability and imprecision in the small heart. Third, there is markedly less mortality, morbidity, and need for inotropic support after stage II, compared to the stage I/Norwood operation. The second stage of the univentricular palliation is a step towards more clinical stability, which is characterised by a strong need for systemic vasodilation. Thus, the natural post-operative improvement of the hemodynamics observed after stage II may confound the detection of functional improvement. Fourth, the primary efficacy results reveal no improvement in cardiac function (fractional area change or strain) at 3 or 12 months. Concerns arise from unfavourable changes in longitudinal strain at 3 months in the treatment group. Although this resolved by 12 months, together with greater post-procedure troponin level increases, these effects suggest myocardial injury from the intramyocardial cell injections. Fifth, the control group did not receive placebo injections. While the treatment dose in 8 × 0.1 mL epicardial injections was 1–3 million total nucleated cells per kg/body weight, based on cell numbers before cryopreservation, conspicuously no information was presented regarding post-thaw yield characterisation of cell types, cell viability and specific cell counts of product actually delivered. Finally, the higher incidence of serious adverse events at 3 months in the treatment group is a safety concern, even if not statistically significant at 12 months. While no serious adverse events were deemed related to the cell product itself, this deserves further scrutiny in future studies.

Instead, our recent phase I clinical trial [2], was the first study to deliver autologous CB-MNCs to 10 neonates with HLHS during the Norwood procedure on post-natal day 2 or 3, providing critical data on the safety and feasibility of an innovative technique for delivering a large number of cells to the myocardium during cardioplegic arrest. An average of 644.9 ± 134 million CB-MNCs per treatment were collected, processed, and tested stringently to NetCord-FACT international standards (Foundation for the Accreditation of Cellular Therapy) [2, 3]. As we previously demonstrated in a large animal model [4], by delivering CB-MNC to the entire coronary vasculature of the very small neonatal heart during cardioplegic arrest, this approach allows for extensive trafficking of cells into the interstitium and myocardial uptake of a very large number of cells without injury risk or cell loss associated with direct myocardial injections or intracoronary infusion in a beating heart.

Most importantly, the timing of cell delivery within the first few days of life during the Norwood operation, was specifically chosen to supplement the neonatal cellular plasticity and potential for myocardial cell hyperplasia persisting at this stage [5, 6]. Other cell therapy trials (which commonly employ multiple epicardial injections) have avoided treating during the Norwood procedure because of the morbidity and mortality risk associated with stage I. In order to ensure minimal cell loss and degradation of the CB-MNC, we avoided cryopreservation by maintaining cells in cold storage, then processing on the day of the operation, delivering high quality, metabolically viable cells.

It is important that cell therapy works synergistically with staged surgical palliation for HLHS, which allows for gradual cardiovascular adaptation to the unique demands of single ventricle physiology. After birth and until stage II, the single RV is powering a circulation in parallel to both systemic and pulmonary vascular beds. For a patient with HLHS, shortly after birth the RV works with a volume loading 3–4 times greater than compared to a normal RV, and the pulmonary-to-systemic blood flow ratio (Qp: Qs) is 2 or 3:1. Post-Norwood, the Qp: Qs may be reduced to 1:1. This still represents RV volume loading that is twice that of the normal RV. This pressure and volume-overloaded state stimulates myocardial growth, involving hyperplasia and cell proliferation [7, 8]. During the stage II operation (bidirectional cavopulmonary shunt or bidirectional Glenn) the superior vena cava is connected directly to the pulmonary arteries, and the systemic-to-pulmonary shunt is removed. The circulatory physiology is now in series, resulting in an immediate unloading of the RV volume by 50% relative to a normal systemic ventricle. Notably, stage II stimulates a reduction of myocardial mass, involving apoptosis [9]. Therefore, the optimal timing for cell therapy is during the Norwood operation to enhance myocardial remodelling processes, and not with stage II.

Beyond timing and delivery, CB-MNC paracrine strategy targets inflammation and fibrosis while also supporting RV myocardial survival by limiting myocyte loss and promoting angiogenesis, thus sustaining myocardial performance. Whilst extracellular vesicles contribute to cell therapy paracrine responses, vesicular intrinsic content and properties arise from specific cell- and condition-dependent local micro-niche interactions between donor cells and myocardium. Thus, identification of the optimal donor cell type requires more investigation. Human pluripotent stem cell-derived cardiac progenitor cells for HLHS were investigated by the TICAP/PERSEUS trials, which reported long-term positive improvements in single ventricle function with lower complication rates following intracoronary cell delivery [10]. Despite design limitations (anatomical and staging heterogeneity, open label, small sample size), these early-phase trials provide important evidence for future work.

Cell-free products manufactured from extracellular vesicles represent an emerging therapeutic modality, administered without direct myocardial injection, promoting paracrine effects and potentially offering advantages in preservation, standardisation, and safety compared to whole cell therapies. However, direct remuscularisation to fortify RV function will require fundamentally different strategies involving direct engraftment with myocytes or engineered tissues manufactured from human induced pluripotent stem cells.

Although this phase IIb clinical trial [1] is a landmark multicenter study of cell therapy for HLHS, clear benefits in cardiac function were absent, at least at 12months follow-up. However, the valuable safety data while not perfect, does not preclude future carefully designed trials. Careful refinement of cell delivery methods, timing of treatment, cell types, cell dosage, demonstration of cell viability, and patient selection with cardiac MRI endpoints, may yet yield positive results; even if a large randomized multicenter trial is an elusive goal.

## Data Availability

No datasets were generated or analysed during the current study.
